# Constructing Offender Typologies From Judicial Data: A Clustering Analysis of Sextortion Cases

**DOI:** 10.1177/10790632261434143

**Published:** 2026-03-12

**Authors:** Fangzhou Wang, Weiping Pei

**Affiliations:** 1 Department of Criminology and Criminal Justice, 12329 University of Texas at Arlington, Arlington, TX, USA.; 2 School of Cyber Studies, 8050 The University of Tulsa, Tulsa, Oklahoma, USA.

**Keywords:** Sextortion, Clustering Analysis, Offender Typology, Victimization

## Abstract

Sextortion is a form of digitally facilitated sexual abuse that causes severe psychological, financial, and social harm, particularly to minors and marginalized adults. This study presents the first quantitative typology of people who commit sextortion, based on a clustering analysis of 111 U.S. judicial cases. Four distinct subtypes of offenders were identified: adult-focused financial sextortion; minor-focused CSAM-related sextortion; technologically sophisticated coercion of minors, sometimes resulting in fatal outcomes; and adult-focused hybrid sextortion that escalates from online manipulation to in-person exploitation. These subtypes differ in motivation, technical capacity, victim profiles, and sentencing outcomes, with the most technologically advanced group paradoxically receiving more lenient sentences. Overall, the findings extend existing typologies and highlight the need for differentiated investigative, prosecutorial, and policy responses to address the varied tactics and harms of sextortion.

## Introduction

Sextortion, a term combining “sexual” and “extortion,” refers to a form of image-based sexual abuse in which people who offend use threats involving sexually explicit material to coerce, manipulate, or control victims. Typically, people who commit sextortion obtain sexual images, videos, or personal information and threaten to distribute these materials unless victims comply with specific demands, which may include providing additional explicit content, engaging in sexual acts, making financial payments, or submitting to ongoing control (Henry & Powell, 2018; McGlynn & Rackley, 2017; Patchin & Hinduja, 2018; Wolak et al., 2018). Although sextortion falls within the broader category of image based sexual abuse (IBSA), it is distinguished from other forms such as nonconsensual pornography or sexualized deepfakes by its reliance on threatened rather than actual dissemination as the primary mechanism of coercion. In many cases images are never publicly released as the offending behavior is oriented toward ongoing control or gain rather than exposure itself (O’Malley, 2025).

People who commit sextortion constitute a heterogeneous group that often employs similar coercive tactics but acts on diverse motivations. Historically, sextortion most commonly involved threats to disclose sexual images unless victims provided additional explicit material (U.S. Immigration and Customs Enforcement, n.d.), yet empirical evidence demonstrates substantial variation in offender intent. Analyses of open-source cases of online sexual coercion and extortion indicate that incidents involving minors are predominantly sexually motivated, with most people who offend seeking additional sexual content or sexual access (Europol, 2017), whereas sextortion involving adults more frequently occurs within the context of intimate partner violence or prior romantic relationships (Eaton et al., 2017, 2023; Wolak & Finkelhor, 2016). More recently, financial sextortion has emerged as a dominant pattern, particularly among adolescents, with reports to the National Center for Missing & Exploited Children more than doubling between 2022 and 2023 and teenage boys aged 15 to 17 disproportionately targeted through peer impersonation and payment demands involving gift cards, wire transfers, or cryptocurrency (Davis, 2023; Vaughan, 2024). Victimization patterns further underscore the diversity of sextortion contexts: while minors are widely recognized as a high-risk group, prevalence studies indicate that sextortion affects both youth and adults, with higher overall rates reported among adults in population-based surveys (Henry & Umbach, 2024; Ray & Henry, 2025). Among minors, gendered patterns are pronounced, with male adolescents more commonly targeted for financial sextortion and female minors more frequently subjected to sexually motivated coercion (Thorn, 2017, 2024b; Wolak et al., 2018). Regardless of age or gender, sextortion is associated with significant harms, including anxiety, depression, shame, fear of exposure, social withdrawal, financial loss, and suicidal ideation, and these consequences often persist beyond the immediate incident, disrupting victims’ personal, social, and professional lives (Henry et al., 2023; Mandau, 2021; Walsh & Tener, 2022).

Despite this heterogeneity in offending patterns and victimization contexts, sentencing responses to sextortion remain fragmented and inconsistent. Because sextortion is rarely prosecuted under statutes specifically designed to address the crime, prosecutors must rely on traditional extortion, computer crime, or child exploitation laws, each of which captures only part of the underlying harm. Consequently, offenders who target minors are frequently prosecuted under federal child exploitation statutes and face lengthy mandatory minimum sentences, whereas offenders who target adults or rely on hacking or coercive threats often receive comparatively minor sentences, even when they victimize hundreds of individuals. This disparity reflects that sentencing outcomes depend more on available charging pathways than on the severity of the coercive sexual conduct itself (Robbins, 2019; Wittes et al., 2016a).

Based on the background literature highlighting the seriousness and prevalence of sextortion among both adults and minors, this study seeks to redirect academic attention to an underexplored issue within sextortion research: the systematic categorization of people who commit sextortion through empirically grounded typologies. Although prior studies have made important contributions to classifying people who engage in sextortion (O’Malley et al., 2023; O’Malley & Holt, 2022), this work has relied primarily on theme-based qualitative methodologies rather than quantitative approaches. This emphasis reflects long-standing data limitations, including restricted access to complete and standardized case records, which have constrained the development of systematically coded datasets and, in turn, large-scale quantitative analysis.

Quantitative methods offer distinct advantages for addressing these limitations. While qualitative research provides critical insight into experiences, motivations, and meanings, it is less suited to assessing whether observed patterns hold consistently across broader populations. The quantitative approach employed in this study, particularly unsupervised clustering, enables the identification of underlying structures in offender behavior without imposing predefined categories. By examining co-occurring behaviors, technologies, and offense characteristics, clustering reveals recurring behavioral configurations that may be difficult to detect through case-by-case qualitative interpretation. In this sense, quantitative modeling complements rather than replaces qualitative research by validating, refining, or challenging existing thematic insights within a scalable and replicable framework.

To address this gap, the present study applies unsupervised clustering analysis to judicial records to identify empirically derived typologies of people who commit sextortion. This approach allows offender characteristics to group organically across cases, offering a systematic means of assessing whether patterns suggested by prior qualitative work consistently emerge in larger samples. Even when similarities are observed, clustering contributes analytic value by clarifying which distinctions are empirically supported and how specific behaviors co-occur within offender subgroups (Garcia-Dias et al., 2020; Hart et al., 2017). Rather than asserting methodological superiority, this strategy aims to strengthen the evidence base by producing replicable offender configurations that can more reliably inform prevention strategies, investigative priorities, and policy responses.

### Sextortion Against Minors

Sextortion against minors has received increasing research and policy attention, particularly as financially motivated sextortion has surged in recent years. Youth-targeted payment demands now represent the fastest-growing sextortion subtype reported to U.S. law enforcement and child-protection organizations, highlighting severe psychological and social harms and ongoing challenges for prevention and investigation (Davis, 2023; FBI, 2024; Thorn, 2025). Empirical studies estimate minor victimization rates between 2.6% and 5.3% and perpetration rates from 0.7% to 3% (Finkelhor et al., 2022, 2024; Gámez-Guadix et al., 2022; Patchin & Hinduja, 2018, 2020, 2024; Ray & Henry, 2025). Research consistently documents overlap between victimization and perpetration in sextortion and related forms of image-based abuse, cyberviolence, and offline delinquency, often attributed to learned behaviors, dysfunctional coping, or retaliatory motives (Chan & Wong, 2020; Clancy et al., 2020; Gámez-Guadix et al., 2023; Patchin & Hinduja, 2020; Posick, 2013).

Multiple factors heighten youth vulnerability. Non-heterosexual adolescents face higher victimization risk (Patchin & Hinduja, 2018, 2024; Ray & Henry, 2025), and while females are more frequently victimized, some studies find males more likely to be both victims and people who perpetrate sextortion (Finkelhor et al., 2024; Gámez‐Guadix et al., 2022; Henry & Umbach, 2024; Patchin & Hinduja, 2018, 2020; Wittes et al., 2016b; Wolak et al., 2018). Findings on age remain mixed (Gámez-Guadix et al., 2022; Patchin & Hinduja, 2020, 2024; Ray & Henry, 2025). Behavioral risks such as sexting substantially increase vulnerability, with occasional sexting associated with a 35% risk and frequent sexting exceeding 55% (Alsoubai et al., 2022; Ray & Henry, 2025). Broader psychosocial factors include desire for peer acceptance, cognitive immaturity, prior sexual experiences, and unstable family environments (Dolev-Cohen et al., 2023). Developmental research further highlights adolescents’ heightened susceptibility due to underdeveloped impulse control, sensation seeking, and limited understanding of privacy and trust (Chou & Chou, 2023; Romer et al., 2017; Silva et al., 2016; Victor & Hariri, 2016), compounded by extensive digital media use (Patchin & Hinduja, 2020; Wolak et al., 2018). Sextortion frequently occurs within teen dating violence and digital dating abuse contexts, where technology is used for coercion and control (CDC, 2025; Patchin & Hinduja, 2020; Wolak et al., 2018; Zweig et al., 2013).

Incident-level research shows that people who commit sextortion are often known to victims, including romantic partners, offline acquaintances, or online contacts (Alsoubai et al., 2022; Henry & Umbach, 2024; Patchin & Hinduja, 2018, 2024; Wolak & Finkelhor, 2016). Threats typically involve sharing explicit material with peers, posting it online, or informing parents, and many people who offend rely on fear rather than executing threats (Edwards & Hollely, 2023; Patchin & Hinduja, 2024; Wolak & Finkelhor, 2016). Preparatory behaviors commonly include catfishing and identity deception (Açar, 2016; Edwards & Hollely, 2023; O’Malley et al., 2023; Wittes et al., 2016b), facilitated by VPNs, phishing, and anonymous platforms (O’Malley et al., 2023; Wittes et al., 2016b). Grooming strategies often involve flattery, fabricated relationships, or posing as authority figures such as modeling agents (Kopecky et al., 2015; O’Malley et al., 2023). Existing typologies distinguish people who offend through grooming, cybercrime-based targeting of multiple victims, intimate partner exploitation, and transnational financially motivated schemes (O’Malley & Holt, 2022).

Across cases, minors commonly experience repeated harassment, significant psychological distress, and chronic underreporting, with some incidents escalating to offline threats or physical victimization (Gámez-Guadix et al., 2022; O’Malley & Holt, 2022; Ray & Henry, 2025). Fear of shame, punishment, and stigma often suppresses disclosure, leading many victims to seek anonymous support and delaying effective intervention (Tangney & Dearing, 2003; Wittes et al., 2016b; Wolak & Finkelhor, 2016).

Overall, the literature on minor-focused sextortion has centered on prevalence, developmental risk factors, offender strategies, and psychological harm, primarily using surveys, interviews, and descriptive methods. While foundational, these approaches leave room for complementary methods capable of capturing dynamic digital behaviors and structural patterns. Integrating systematic quantitative or computational approaches with qualitative insights may strengthen evidence-based prevention and intervention. As the next section shows, similar methodological gaps exist in research on adult sextortion, underscoring the need for scalable, integrative approaches across life stages and contexts.

### Sextortion Against Adults

The literature on adult targeted sextortion describes a multifaceted and evolving crime shaped by demographic, psychological, technological, and cultural factors. Although research remains limited, studies identify multiple pathways including hacking into devices or webcams, exploiting consensually or nonconsensually shared sexual images, misusing materials exchanged within intimate relationships, and manipulating victims through online romance scams and fake profiles (Paat & Markham, 2021; Whitty, 2015). Compared to minors, adult sextortion more frequently involves financial extortion and unfolds within broader life contexts (Brighi et al., 2023; O’Malley & Smith, 2025; Ray & Henry, 2025; Tzani et al., 2024; Wolak et al., 2018).

While adult victims are predominantly female, gender disparities are narrower than in youth cases. Victimization is most common among adults aged 18 to 34, and disproportionately affects men, sexual minorities, and racial minorities including Black and Native American women (Cross et al., 2023; Eaton et al., 2023; Henry & Umbach, 2024; Ray & Henry, 2025). Men are more often targeted for financial extortion through fraudulent romantic connections, whereas women are more likely to face coercion involving hacked or shared images, sexual demands, or reputational threats (Mondal et al., 2022). Sexual minorities experience especially high victimization and psychological distress and are less likely to seek formal help due to stigma and fear of exposure (Gohil & Fido, 2021; Miguel-Alvaro et al., 2024; Ray & Henry, 2025).

Adult sextortion is frequently embedded within intimate partner violence. Prior intimate partner violence predicts subsequent sextortion particularly during crisis periods such as the COVID 19 pandemic when shared images are weaponized to maintain control (Ross et al., 2019; Bates, 2017; Eaton et al., 2023). Nonconsensual pornography has been conceptualized as a form of intimate partner violence involving coercion and intimidation (Eaton et al., 2023), and prior victimization increases vulnerability to revictimization reflecting broader patterns of polyvictimization (Carbone-Lopez et al., 2012; DeKeseredy et al., 2019; Hamby et al., 2020; Krebs et al., 2011; Smith et al., 2003).

People who commit sextortion are often strangers or former or current romantic partners, and some overlap exists between victim and offender populations particularly in certain cultural contexts (Eaton et al., 2023; Henry & Umbach, 2024). Psychological risk factors for adult victims include emotional instability, attachment related anxiety, low conscientiousness, and elevated sexual need, all of which predict vulnerability in quantitative models (Tzani et al., 2024). Victim responses range from panic and compliance to blocking or reporting, yet repeated extortion is common and recovery often depends on social support. Suicidal ideation is alarmingly prevalent with shame humiliation and depressed mood as key predictors (Brighi et al., 2023; Gohil & Fido, 2021; Howard & Rokach, 2017; Miguel-Alvaro et al., 2024; O’Malley, 2023).

Sextortion typically follows a trajectory of grooming image sharing and extortion resulting in lasting psychological harm including anxiety depression and social withdrawal (Wang, 2024). Although adults are somewhat more likely than minors to seek help, formal reporting remains rare due to stigma, distrust of authorities, fear of victim blaming, and limited legal recognition of sextortion in many jurisdictions (O’Malley, 2023; O’Malley & Smith, 2025). In some countries, the absence or underdevelopment of sextortion-specific legislation further restricts legal recourse (O’Malley & Holt, 2022; Wittes et al., 2016a). Cultural norms and gendered expectations intensify harm with some victims, particularly women experiencing heightened blame and inadequate justice system responses including lenient sentencing and weak institutional support (Alemi et al., 2025; O’Malley & Holt, 2022; Ray & Henry, 2025).

In sum, adult sextortion is characterized by greater financial extortion, broader demographic diversity, and complex relational dynamics often intersecting with intimate partner violence and online deception. While adult- and minor-focused studies share challenges such as underreporting and repeated victimization, adult cases reflect wider social and contextual variation. Despite these advances, the literature remains constrained by reliance on self-reported and cross-sectional data. Future research should integrate advanced quantitative methods including clustering and predictive modeling to identify latent victim profiles, examine cross group differences, and test theories of risk and resilience across the life course.

## Current Study

Building on the foundational inquiry of O’Malley and Holt (2022) on cyber sextortion typologies of people who commit sextortion, this study advanced by applying a complementary methodological approach using a different dataset. While O’Malley and Holt’s (2022) research provided critical insights through qualitative content analysis and inductive thematic coding, identifying four prominent categories of people who offend, including minor-focused, cybercrime-based, intimately violent, and transnational financial actors, the present study adopts a quantitative, computational approach grounded in unsupervised machine learning. Specifically, clustering analysis is employed to examine judicial case data with the objective of empirically identifying groupings of people who commit sextortion and assessing whether similar patterns emerge within a new, systematically structured dataset. This approach seeks to extend and enrich existing knowledge by offering a statistically supported typology that complements and builds upon the conceptual contributions of prior research.

A central objective of this study is to examine whether the application of clustering analysis to judicial data will yield typologies of people who commit sextortion consistent with those identified by O’Malley and Holt (2022), or whether additional/different groupings will emerge. The study’s contribution is twofold. First, it offers methodological innovation by introducing scalable, replicable statistical modeling to the study of classification of people who commit sextortion, a domain historically dominated by narrative and thematic analysis. Second, it provides actionable insights for both investigators and policymakers by identifying profiles of people who commit sextortion grounded in real-world judicial evidence. These empirically derived typologies may help inform targeted prevention strategies, enhance detection protocols, and improve prosecutorial approaches by linking behavioral traits to specific categories of people who commit sextortion.

## Method

### Ethics

This study relied exclusively on publicly available federal judicial documents and official press releases pertaining to sextortion cases in the United States. No direct interaction with human participants occurred, nor were any identifiable private or sensitive data collected. Consequently, formal review by an Institutional Review Board (IRB) or equivalent ethics committee was not required. All materials analyzed were obtained from open government sources and were already in the public domain. Because the research involved only secondary data of this nature, the requirement for informed consent was not applicable.

### Data

A total of 111 sextortion cases were identified through a two-stage search and verification process using U.S. Department of Justice (DOJ) press releases, with each case corresponding to a unique DOJ source. An initial keyword search for “sextortion” yielded 1,152 results, which were systematically screened to identify federal criminal cases involving sextortion-related conduct. Inclusion criteria required that cases involve an explicit threat to distribute sexually explicit images or videos unless the victim complied with specific demands, and that the offense be carried out through a digital medium (e.g., social media, email, messaging applications, or video platforms). Cases involving threats made solely through offline or face-to-face interactions were excluded. Many search results involved child pornography production or possession and were excluded unless court documents explicitly documented the use of threats to distribute sexual content to obtain further compliance (e.g., additional images, sexual acts, financial payment, or behavioral control). This approach ensured analytical clarity by isolating cases meeting the definitional threshold of cyber sextortion rather than conflating them with broader forms of child exploitation. After removing duplicates, non-criminal announcements, and cases lacking sufficient detail, the final dataset comprised 111 unique individuals and corresponding DOJ case sources. Consistent with best practices for research transparency, we report our sample size determination, data exclusions, and all measures used in the study (Nelson et al., 2012).

Following identification, each person’s name was cross-referenced in the PACER Monitor database to locate corresponding federal court documents and construct a more detailed case profile. For each case, the primary sources used for coding were the sentencing memoranda submitted by prosecutors or defense attorneys and/or the original indictments. These documents were prioritized due to their richness in factual background, including detailed narratives of the person’s conduct, motivations, victim characteristics, and offense methodology. In cases where certain files were unavailable or incomplete, alternative filings such as the criminal complaint or docket summary were used to supplement missing information.

### Variables Coding and Selection

In this study, we extracted 17 variables from qualitative judicial documents to support a clustering analysis aimed at identifying distinct patterns among sextortion-related cases (see Table 1). Variable selection was grounded in established scholarship on sexual offending, online harassment, cyber-enabled coercion, and stalking risk factors (e.g., DeKeseredy et al., 2019; Lussier et al., 2024; Patchin & Hinduja, 2020; Sparks, 2021). All variables were coded using a structured codebook by two independent coders who reviewed the same sentencing documents. Discrepancies were resolved through multiple consensus meetings. Interrater reliability, assessed using Cohen’s kappa, yielded a mean value of 0.82, indicating substantial agreement.

**Table 1.** Descriptive Table for All Variables

Variable name	Coding type	Coding (0 = no, 1 = yes)	Operational definition
Sentencing outcomes	Continuous	Numbers of months	Length of prison sentence assigned to the person who offended
Prior criminal history	Binary	0/1	Whether the person who offended had any documented prior convictions or criminal record
Creation of fake identity	Binary	0/1	The person who offended used a false persona or alias to communicate with victims
Using hacking tools	Binary	0/1	Evidence of account intrusion, malware use, password cracking, or unauthorized digital access
Making financial demands	Binary	0/1	Person who offended demanded money, gift cards, wire transfers, or cryptocurrency
Making sexual demands	Binary	0/1	Requests for additional explicit photos, videos, or sexual acts
Requesting to meet in-person	Binary	0/1	Any attempt to arrange a face-to-face meeting with the victim
Received demanded money	Binary	0/1	Person who offended successfully obtained money from the victim
Victim death	Binary	0/1	The case resulted in the victim’s death (e.g., suicide)
Minor victims	Binary	0/1	The victims in the case were minors (younger than 18)
Distribution of explicit materials	Binary	0/1	Distribution of explicit images/videos of victims (adult victims)
Production of explicit materials	Binary	0/1	Person who offended compelled victims to produce new explicit content
Involvement of other crimes	Binary	0/1	Involvement in two or more non-sextortion offenses
Fraud and financial crime	Binary	0/1	Evidence of fraud committed online, including identity misuse or scams
Cyberstalking and online harassment	Binary	0/1	Repeated threats, intimidation, or stalking via digital platforms
Computer and identity crimes	Binary	0/1	Unlawful access, identity theft, or misuse of personal information
Production/distribution/possession of CSAM	Binary	0/1	Person who offended produced, distributed, or possessed child sexual abuse material

#### Legal Process Variables

Two variables capture legal-process characteristics relevant to offender behavior and case outcomes. *Sentencing outcome (months)* records the total custodial sentence imposed, allowing assessment of whether clusters correspond to meaningful differences in sentencing severity. Prior research shows that sentencing outcomes in sexual-related offenses are influenced by victim age, coercive tactics, and offense escalation (Amirault & Beauregard, 2014; Beeby et al., 2021; Robbins, 2019; Rydberg et al., 2018; Wittes et al., 2016a). *Prior criminal history* (0/1) indicates whether the offender had any documented criminal record. Prior offending has been linked to greater persistence, elevated coercion, and distinct risk profiles, making this variable important for understanding offender trajectories across clusters (Lussier et al., 2010, 2024; Xiaohan et al., 2025).

#### Offender Tactics and Coercive Methods Variables

Five variables capture core sextortion tactics consistently documented in the legal materials and emphasized in the literature. *Creation of fake identity* (0/1) reflects whether the offender used fabricated or deceptive personas to facilitate anonymity, manipulation, and trust-building (Cross & Layt, 2022; Kennedy et al., 2021; Wall, 2017). *Using hacking tools* (0/1) captures the deployment of malware, unauthorized access techniques, or password-cracking tools to obtain sensitive information, distinguishing technologically sophisticated offenders from those relying solely on interpersonal manipulation (O’Malley & Holt, 2022; Patchin & Hinduja, 2020; Ray & Henry, 2025). *Making financial demands* (0/1) indicates explicit requests for money or assets, reflecting financially motivated coercion commonly accompanied by reputational threats (O’Malley & Holt, 2022; Tzani et al., 2024; Wang, 2025). This variable is analytically distinct from *receiving demanded of payment* (0/1), which captures whether a financial transfer actually occurred. Extortion research emphasizes that successful payment reflects greater offender effectiveness, persistence, and coordination, marking a more escalated offending pattern (Brown et al., 2025; Estévez-Soto et al., 2021; Levi, 2017). *Making sexual demands* (0/1) captures requests for sexual images, acts, or explicit compliance, reflecting motivations rooted in sexual coercion and exploitation rather than purely financial objectives (O’Malley & Holt, 2022; Seto, 2019, 2021). *Requesting to meet in person* (0/1) identifies attempts to escalate online coercion into offline encounters, a trajectory associated with elevated victim risk in cyberstalking and online grooming research (Jeglic & Winters, 2023; Spidell et al., 2021; Weekes et al., 2025).

#### Victim-Related and Harm-Related Variables

Victim- and harm-related variables capture incident severity and key risk factors. *Victim death* (0/1) records whether the victim died by suicide or other causes during or after the extortion, reflecting extreme harm outcomes emphasized in sextortion, cyberbullying, and online harassment research (Collantes et al., 2020; Nilsson et al., 2019; O’Malley & Smith, 2025; Rodway et al., 2023). *Minor victims* (0/1)^1^ indicates the presence of at least one victim under age 18. Although judicial documents rarely specify exact ages, the involvement of minors consistently emerged as a critical marker of case severity. This binary measure captures youth involvement when precise age data are unavailable and reflects the heightened legal and psychological significance of minor victimization in sextortion cases (O’Malley et al., 2023; Patchin & Hinduja, 2020). *Distribution of explicit materials *(0/1) captures whether sexual images were disseminated, representing a major escalation mechanism that amplifies harm by extending victimization to broader audiences (Bloxsom et al., 2024; Broadhurst, 2019; O’Malley & Holt, 2022). *Production of explicit materials* (0/1) indicates the creation of new coerced sexual content, signaling a more intrusive and exploitative form of offending distinct from content dissemination (Cale et al., 2021; Rimer, 2019; Salter & Wong, 2024).

#### Additional Offense-Related Variables

These variables capture co-occurring criminal behaviors reflecting broader multimodal offending patterns. *Involvement of other crime* (0/1) indicates whether sextortion occurred alongside at least one additional non-sextortion offense, functioning as a higher-level indicator of offense multiplicity rather than offense type. *Fraud and financial crime* (0/1) captures manipulation-based financial wrongdoing, such as social engineering or romance fraud elements, distinct from technical intrusions. *Cyberstalking and online harassment* (0/1) identifies persistent or escalating threats, intimidation, or coercive communication beyond an initial demand, distinguishing sustained harassment from isolated incidents. *Computer and identity crimes* (0/1) capture unauthorized system access or identity misuse, reflecting technical rather than interpersonal offending. Finally, *production, distribution, or possession of CSAM* (0/1) identifies formal child sexual abuse material charges, distinguishing offenders whose conduct extends into established child exploitation categories and more severe offending trajectories.

## Analysis

### Factor Analysis of Mixed Data (FAMD)

Prior to clustering, we applied dimensionality reduction to preserve meaningful variance while reducing noise and improving interpretability. Because the dataset includes both categorical and continuous variables, we employed Factorial Analysis of Mixed Data (FAMD), which is designed for mixed-type datasets and allows both variable types to be analyzed within a single framework (Lê et al., 2008; Pagès, 2014).

The dataset consists of 111 observations and 17 variables, yielding more than six observations per variable, which is adequate for exploratory dimensionality-reduction procedures. Dimensions were retained based on explained variance and interpretability. We initially considered the number of victims as a potentially relevant variable; however, its contribution to the retained dimensions was negligible, and its inclusion did not affect cluster assignments. Accordingly, this variable was excluded from the clustering procedure. Nevertheless, We report the average number of victims by cluster for descriptive context.

### Dimension Retention and Variable Contributions

To determine the number of dimensions to retain, we examined the distribution of explained variance across components (see Table 2). The first five dimensions together accounted for 62.31% of the total variance, capturing the primary structure of the data while limiting redundancy. On this basis, five dimensions were retained for subsequent clustering.

**Table 2.** Eigenvalues and Percentage of Explained Variance for the First Five Components From the FAMD

Component	Eigenvalue	% of variance	% of variance (cumulative)
0	27.953	21.67%	21.67%
1	16.127	12.50%	34.17%
2	14.148	10.97%	45.14%
3	11.784	9.13%	54.27%
4	10.365	8.03%	62.31%

To aid interpretation, we examined variable contributions across the retained dimensions. Variables exceeding the average contribution threshold on at least one dimension were considered substantively meaningful. As shown in Table 3, financial demands and receipt of demanded money loaded strongly on Dimension 0, victim death on Dimension 1, and distribution of explicit materials on Dimension 4. These patterns indicate that the retained dimensions capture distinct latent structures related to financial coercion, severe harm, and content dissemination.

**Table 3.** Variables With Significant Contributions to the FAMD Dimensions

Variable	0	1	2	3	4	Mean weight
Distribution of materials	-	-	-	-	0.23	0.047
Financial demands	0.352	-	-	-	-	0.074
Involvement of other crimes	-	-	-	0.201	-	0.042
Other crimes: Production, distribution, and possession of child sexual abuse material	0.232	-	-	-	-	0.055
Victim death	-	0.217	-	-	-	0.048
Received demanded money	0.302	-	-	-	-	0.063

### Clustering

**Figure 1.**
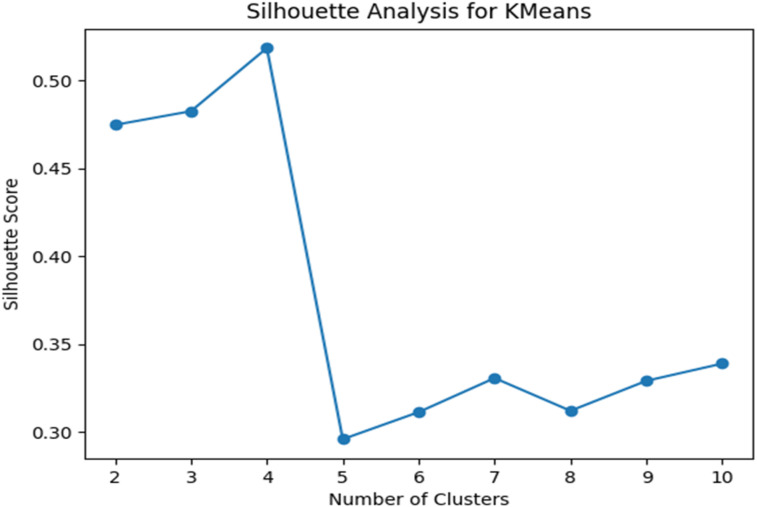
Silhouette scores for K-means clustering with varying numbers of clusters

Clustering was conducted using K-means applied to the reduced-dimension FAMD space. The optimal number of clusters was determined using silhouette scores across solutions ranging from two to ten clusters. As shown in Figure 1, the silhouette score peaked at four clusters (0.5185), indicating adequate separation and internal cohesion. A four-cluster solution was therefore retained for all subsequent analyses. The authors take responsibility for the integrity of the analyses and made every effort to avoid inflating statistically significant findings.

## Cluster Solution Overview

The clustering analysis produced a four-cluster solution. The identified subtypes include: *Adult-Focused Financial Sextortion offenders*, characterized by opportunistic, low-technology financial manipulation; *Minor-Focused CSAM Sextortion offenders*, involving static, image-based offenses with minimal interpersonal interaction; *Minor-Focused Tech Facilitated Sextortion offenders*, the most technologically advanced and harmful subtype, marked by severe psychological control and fatal outcomes; and *Adult-Focused Tech Escalating Hybrid Sextortion offenders*, which combines high technical skill with escalation from online coercion to in-person contact. Table 4 presents the frequency and relative prevalence of key variables across clusters compared to the full sample to facilitate interpretation of these profiles.

**Table 4.** Distribution of Variables Across Identified Clusters

	Cluster 1 (*N* = 10)		Cluster 2 (*N* = 87)		Cluster 3 (*N* = 4)		Cluster 4 (*N* = 10)	
	*N*	Diff^2^	*N*	Diff	*N*	Diff	*N*	Diff
Sentencing outcomes	-	−0.82	-	0.21	-	−0.11	-	−0.95
Prior criminal history	4	−0.05	39	−0.002	1	−0.20	6	0.15
Creation of fake identity	8	0.26	49	0.02	3	0.21	0	−0.5
Using hacking tools	0	−0.14	4	−0.09	3	0.61	8	0.66
Making financial demands	10	0.76	8	−0.15	4	0.76	5	0.25
Making sexual demands	10	0.04	86	0.03	1	−0.71	10	0.04
Requesting to meet in-person	1	−0.13	21	0.02	0	−0.23	3	0.07
Received demanded money	8	0.61	5	−0.13	3	0.56	5	0.31
Victims death	2	0.15	0	−0.05	3	0.71	0	−0.05
Minor victims	2	−0.60	80	0.13	4	0.21	2	−0.6
Distribution of explicit materials	5	−0.09	48	−0.04	4	0.41	9	0.31
Production of explicit materials	7	−0.16	77	0.03	2	−0.36	9	0.04
Involvement of other crimes	10	−0.08	86	0.01	4	0.02	10	0.02
Online fraud	8	0.73	0	−0.07	0	−0.07	0	−0.07
Cyber-harassment	3	0.06	16	−0.06	4	0.76	4	0.16
Computer/identity fraud	1	0.10	0	−0.10	0	−0.10	10	0.91
Production/distribution/possession of CSAM	1	−0.61	74	0.14	4	0.29	0	−0.71

### Cluster Profiles

#### Cluster 1: Adult-Focused Financial Sextortion Offenders

The first cluster comprises 10 people who offended who primarily target adult victims and engage in digital extortion involving moderate levels of deception and coercion. Statistically, 80% of the victims in this group are adults, indicating a strong preference for non-minor targets. The cluster shows a sentencing diff of −0.82, indicating substantially lighter punishments compared to the overall average. This suggests that courts may have viewed these cases as less severe, possibly due to the low involvement of minor victims, which accounts for only two cases with a difference score of −0.60, and relatively limited fatal outcomes (diff = 0.15). Only four individuals in this group had prior criminal history, yielding a diff of −0.05, suggesting a background of unlawful behavior but not consistent recidivism. On average, individuals in this cluster targeted 22.9 victims.

This behavioral pattern is further supported by moderate use of deception, with eight people who offended creating fake identities (diff = 0.26). However, the group employed virtually no advanced technical means, as none of the people who offended used hacking tools (diff = −0.14). Computer or identity fraud was rare, observed in only one case with a diff of 0.10, and cyber-harassment occurred in just three cases (diff = 0.06), indicating limited persistent digital aggression. This group is clearly distinguished by its dominant focus on financial exploitation. Ten people who offended made financial demands, with a diff of 0.76, and eight were involved in financial crimes such as fraud, yielding a diff of 0.73. Moreover, eight people who offended successfully received the money they demanded, with a diff of 0.61, suggesting that their financial-based schemes were not only coercive but also often effective. Although 10 people who offended also made sexual demands (diff = 0.04), only one requesting to meet the victim in person, reflected in a diff of −0.13. CSAM involvement was minimal in this cluster, with only one person who offended involved in production, distribution, or possession of such material (diff = −0.61), and distribution or production of other explicit materials was similarly low, with five and seven people who offended involved respectively (diff = −0.09 and −0.16).

#### Cluster 2: Minor-Focused CSAM Sextortion Offenders

The second cluster is defined by people who offended whose behaviors center on the exploitation of minors, primarily through content-based offenses rather than interactive or coercive manipulation. There is a total of 87 people who offended in this cluster. An overwhelming 91.95% of victims in this cluster are underage, with 80 documented cases and a diff of 0.13, highlighting a strong preference for minor victims. Reflecting the severity of these crimes, the cluster received the most substantial sentencing among all groups, with a sentencing diff of 0.21. Courts appear to treat these offenses with particular gravity, likely due to the persistent victimization inherent in the continued circulation of CSAM. This cluster involved an average of 41 victims.

Despite this, people who offended in this group are not typically recidivists. Thirty-nine had prior criminal history, but the difference score is at −0.002, suggesting their records are not substantially different from the overall sample. Their tactics do not rely heavily on deception or technology. While 49 people who offended created fake identities, the difference score is only 0.02, and only 4 used hacking tools (diff = −0.09). Cyberstalking or harassment was reported in 16 cases, showing a slight underrepresentation with a diff of −0.06. Computer or identity fraud was completely absent from this cluster (*N* = 0, diff = −0.10), further emphasizing the static and non-technical nature of their offenses.

Interpersonal coercion was also minimal. 86 people who offended made sexual demands (diff = 0.03), and 21 requested in-person meet-ups (diff = 0.02), although the actual rates were lower than in other clusters. Only five people who offended successfully received money from victims, showing a negative diff of −0.13. There were no recorded victim deaths in this cluster (diff = −0.05), and financial motives were rare, with eight people who offended involved in financial crimes (diff = −0.07). What distinguishes this cluster most clearly is its total engagement in CSAM offenses. 74 cases involved production, distribution, or possession of CSAM (diff = 0.14), and people who offended were often involved in production (*N* = 77, diff = 0.03) and distribution (*N* = 48, diff = −0.04) of explicit materials.

#### Cluster 3: Minor-Focused Tech-Facilitated Sextortion Offenders

Cluster 3 represents the most lethal and technologically sophisticated group of people who offended in the dataset. A total of four people who offended can be revealed in this cluster. These individuals exclusively target minors, with all four victims in this cluster being underage (diff = 0.21). They are responsible for the highest rate of victim fatalities, with three of the four cases resulting in death, yielding a difference score of 0.71. Despite this level of harm, sentencing outcomes remain below the dataset average, with a diff of −0.11, ranking second most severe after Cluster 1. This discrepancy may reflect legal complexities, plea negotiations, or difficulties in proving the full scope of their conduct. Only one person who offended in this cluster had prior criminal history (diff = −0.20), suggesting that while some may be new to the justice system, their behaviors are deliberate and premeditated. Individuals in this cluster targeted an average of 62 victims.

People who offended in this cluster display high levels of technical skill and deception. Three people who offended used hacking tools, resulting in a difference score of 0.61, and three also created fake identities (diff = 0.21). All four people who offended engaged in cyber-harassment, reflected in a difference score of 0.76. These people who offended are motivated by both financial and sexual aims. All four made financial demands (diff = 0.76), and three succeeded in receiving money (diff = 0.56). In contrast, sexual demands were made in only one case (diff = −0.71), and none requesting to meet the victim in person (diff = −0.23).

Additionally, their involvement in explicit content distribution is considerable, with four people who offended involved in both the distribution of illicit materials (diff = 0.41) and CSAM-related offenses (diff = 0.29), though CSAM was not the primary focus. Two cases involved the production of explicit materials (diff = −0.36), and four people who offended were involved in other crimes (diff = 0.02).

#### Cluster 4: Adult-Focused Tech-Escalating Hybrid Sextortion Offenders

Cluster 4 includes 10 people who offended who display high levels of technical proficiency and a clear pattern of escalation from digital coercion to in-person exploitation. While the majority of victims in this cluster are adults (80%), there are also two minor victims (diff = −0.60). Despite their advanced behaviors, people who offended in this group received the lightest average sentencing across all clusters, with a sentencing difference score of −0.95. This leniency may be attributable to the relative absence of physical harm or fatalities, as only one case resulted in victim death (diff = −0.05). Cases in this cluster involved an average of 84.7 victims.

These people who offended demonstrate the highest prevalence of prior criminal history among all clusters, with six having documented past convictions (diff = 0.15), indicating entrenched unlawful behavior. Technically, they are highly capable. Eight people who offended used hacking tools, resulting in a difference score of 0.66, and 10 people who offended committed computer or identity fraud, yielding a substantial difference score of 0.91. Notably, none of the people who offended in this group created fake identities (diff = −0.50), opting instead to exploit direct technical compromise.

Their offenses are marked by dual extortion tactics, with five people who offended making financial demands (diff = 0.25) and 10 people who offended making sexual demands (diff = 0.04). All five people who offended also received the money they demanded (diff = 0.31), and three requested to meet the victim in person (diff = 0.07), indicating a pattern of online-to-offline escalation. Although their involvement in CSAM is negligible (*N* = 0, diff = −0.71), they were frequently involved in producing and distributing other explicit materials, with nine cases each (diff = 0.04 and 0.31, respectively).

## Discussion

Prior research on sextortion has largely relied on qualitative accounts emphasizing offender narratives or victim characteristics. The present study advances this literature by applying quantitative, unsupervised clustering to 111 judicially processed cases to identify underlying subtypes of people who commit sextortion based on behavioral, technological, and legal attributes. The analysis revealed substantial heterogeneity, distinguishing offenders by victim age, coercive strategy, technical competence, and sentencing outcome. Four clusters of offenders were identified: (1) Adult-Focused Financial Sextortion, (2) Minor-Focused CSAM Sextortion, (3) Minor-Focused Tech Facilitated Sextortion, and (4) Adult-Focused Tech Escalating Hybrid Sextortion. These clusters span a continuum from low-skill digital extortion to highly sophisticated offending that escalates into physical-world harm.

Unlike prior typologies derived from qualitative thematic analysis, this study offers an empirically verified framework that translates judicial narratives into quantifiable indicators enabling systematic comparison across cases. Although cyber-enabled sexual offenses share common features, the clustering model isolates consistent operational patterns within each group, providing practical markers for investigative profiling, risk assessment, and digital evidence prioritization.

The findings also extend prior frameworks by identifying two underexamined subtypes: minor-focused technology-facilitated sextortion and adult-focused tech-escalating hybrid sextortion. These groups highlight distinct escalation pathways, including intensified technological control associated with severe victim harm and transitions from online coercion to offline exploitation. In doing so, the results both corroborate and refine existing typologies, including those proposed by O’Malley and Holt (2022), while demonstrating that technical proficiency operates as a cross-cutting mechanism enabling both sexual and financial exploitation across victim groups.

### Adult-Focused Financial Sextortion Offenders

Prior research shows that sextortion targeting adults is more likely to involve financial demands and extortion-driven threats, whereas minor-focused cases are more often driven by sexual gratification and content-based exploitation (Henry et al., 2020; Pacheco et al., 2019; Powell & Henry, 2019; Ray & Henry, 2025; Thorn, 2024; Vaughan, 2024). Our findings mirror this distinction, particularly in Cluster 1 and Cluster 4, which represent adult-focused trajectories centered on instrumental financial gain. People who offended in these clusters exhibited high rates of monetary demands and payment receipt, reflecting a pragmatic, outcome-oriented approach. Cluster 1 offenders relied on deceptive identities and low-level digital coercion, consistent with an opportunistic and largely reactive pattern of exploitation, while Cluster 4 offenders combined financial motives with higher technical capacity, including hacking and identity fraud. Overall, these clusters highlight a distinct adult-targeted sextortion pathway characterized by instrumental rationality and operational efficiency, reinforcing prior conceptualizations of adult sextortion as qualitatively different from minor-focused offending.

### Minor-Focused CSAM Sextortion Offenders

The observed cases in this cluster are characterized by a predominance of content focused behaviors involving the possession, production, and distribution of child sexual abuse material, with less evidence of dynamic or escalating offending patterns in the available data. These cases involve overwhelming minor victimization and extensive CSAM activity but minimal interpersonal coercion, identity deception, or technical manipulation, distinguishing them from other sextortion related clusters that rely on hacking, impersonation, or prolonged psychological pressure. Because the dataset does not contain reliable indicators of internal psychological states, the analysis does not permit definitive conclusions about offenders’ underlying motives. Rather than inferring intent, this cluster is best interpreted as a behaviorally distinct configuration within sextortion related federal cases, marked by high volumes of CSAM involvement and stable offense methods over time.

This behavioral concentration reflects a form of online sexual offending in which harm is generated not through direct contact or overt coercion but through the large-scale creation, circulation, and persistence of exploitative material. Although these offenses lack physical proximity, the judiciary increasingly recognizes the profound and enduring psychological and systemic harms associated with CSAM, particularly the ongoing victimization inherent in repeated distribution and consumption. Consistent with this legal framing, individuals in this cluster received the most severe sentencing outcomes in the sample, a pattern best explained by statutory mandatory minimums and legal thresholds tied to CSAM production and distribution rather than evidence of a qualitatively distinct motivational subtype.

### Minor-Focused Tech-Facilitated Sextortion Offenders

The third cluster, although numerically small, represents the most dangerous and technologically advanced subgroup in the dataset. People who offended in this group do not rely on deception alone but actively weaponize digital technologies to exert sustained psychological control and, in some cases, fatal harm. Their behavioral profile is consistent with preferential or sadistic people who offend by engaging in technologically facilitated sexual violence, making this cluster the highest-priority and most alarming subtype identified. This group is uniquely marked by extreme lethality: 75% of cases involved the death of minor victims, making it the only cluster in which digital coercion escalated to physical death. This finding provides rare empirical support for prior research linking sextortion to severe outcomes such as suicide and enduring psychological harm by demonstrating a direct connection between technologically mediated coercion and fatal real-world consequences (Nilsson et al., 2019; O’Malley, 2023, 2025).

People who offended in this cluster exhibit advanced technical proficiency, including frequent use of hacking, fake identity creation, and cyber harassment. These tools function not merely as access mechanisms but as instruments of coercion, surveillance, and behavioral control, enabling prolonged domination through repeated threats, impersonation, and continuous monitoring. Unlike the static, content-focused offending observed in Cluster 1, this group displays sustained technological escalation, transforming sextortion into a dynamic, transactional process. Although prior studies have documented individual elements such as impersonation, threats, and coercive surveillance (O’Malley et al., 2023; O’Malley & Holt, 2022; Patchin & Hinduja, 2020, 2024; Walsh & Tener, 2022; Wittes et al., 2016b), the present findings integrate these behaviors into a cohesive empirical profile. This cluster also diverges from traditional minor-focused models by emphasizing financially motivated coercion rather than material accumulation or direct sexual exploitation, reflecting a growing trend in financially driven sextortion targeting minors (Greening, 2025; O’Malley, 2023; Thorn, 2024b). This shift is particularly concerning given minors’ heightened vulnerability to psychological distress and suicide risk due to ongoing neurological development and limited emotional regulation (Chou & Chou, 2023; Romer et al., 2017; Victor & Hariri, 2016).

### Adult-Focused Tech-Escalating Hybrid Sextortion Offenders

This cluster represents a digitally sophisticated, contact-oriented typology marked by a clear trajectory from online manipulation to physical exploitation. Compared to other clusters, it shows the strongest tendency to escalate harm beyond the digital realm: approximately 30% of people who offended made explicit in-person demands, the highest proportion observed, and 100% issued direct sexual demands, indicating an immediate intent to exert sexual control over victims.

These patterns reflect a hybrid form of sextortion in which digital tools are used for access, grooming, and coercion, but the primary objective is in-person sexual exploitation rather than sustained online control or purely financial gain. Although this cluster shares certain online tactics with Cluster 1, their motivations diverge sharply. Whereas Cluster 1’s sexual demands are embedded within financially motivated extortion, the present cluster is driven by sexual exploitation, underscoring the importance of distinguishing sexually motivated from financially motivated sextortion in investigative and preventive contexts.

This cluster is further characterized by high behavioral adaptability and long-term risk. All individuals in this group had prior criminal histories, the highest rate across clusters, and many appear to have progressed from earlier offenses such as fraud, identity theft, or digital harassment into more complex sextortion schemes. Their ability to move fluidly between virtual coercion and offline exploitation, combined with procedural familiarity and risk tolerance, aligns with characteristics of career or enterprise-type people who offend. In contrast, Cluster 3 (minor-focused technology-facilitated sextortion) exhibited lower prior conviction rates despite greater technological sophistication and lethality, suggesting a more covert pathway in which early offending went undetected. Despite the escalation risk observed in the present cluster, sentencing outcomes were relatively light, highlighting the need for more nuanced prosecutorial and prevention strategies that account for hybrid trajectories bridging digital coercion and physical exploitation.

## Limitations

Despite the strengths of applying unsupervised machine learning to judicial sextortion cases, several limitations should be noted. *First*, the dataset relies on publicly available federal cases from the U.S. Department of Justice and PACER Monitor, which likely overrepresent severe or high-profile incidents while excluding state-level, lower-profile, and unreported cases. Because sextortion is not a standalone federal offense and searches relied on the term “sextortion,” cases prosecuted under other statutes, particularly those embedded in intimate partner violence or financial extortion, may be underrepresented. Future research should incorporate state-level records, broader statutory searches, and multiple data sources. *Second,* judicial records contain inherent information gaps. Variables were coded as present only when explicitly documented in the court materials, and information that was not mentioned was treated as missing rather than assumed absent. Even with this conservative approach, reliance on complaints, indictments, and sentencing documents makes it difficult to distinguish true absence from undocumented presence, particularly for variables such as prior criminal history and relational context that are reported inconsistently and are not uniformly corroborated across cases. This limitation reflects a broader constraint of court-based data and introduces potential measurement uncertainty that should be considered when interpreting the findings. *Third*, the sample size of 111 cases reflects persistent data-availability constraints in sextortion research, which often overlaps with related offenses such as CSAM and nonconsensual image distribution. As such, the modest sample size should be understood as a reflection of broader empirical challenges in obtaining comprehensive and disaggregated sextortion datasets rather than as a methodological shortcoming unique to this study. Larger, integrated datasets combining judicial, law-enforcement, and platform-level data are needed to support broader generalization, temporal analyses, and cross-national comparisons. *Fourth,* the modest sample size limits clustering stability and generalizability. Although dimensionality reduction mitigated overfitting, this study represents an initial application of unsupervised clustering to judicial sextortion cases, and larger samples are needed to refine and validate the typology*. Fifth*, clustering was based on observable behaviors rather than offender motivation, as judicial records emphasize legally relevant conduct over psychological intent. Accordingly, the clusters should be interpreted as behaviorally derived configurations rather than motivational typologies. Future work integrating court data with supplemental sources may clarify how motivation intersects with behavioral patterns. *Sixth*, although cluster solutions ranging from two to 10 were systematically evaluated and the four-cluster solution was selected based on the highest Silhouette Score (0.5185), the resulting clusters are imbalanced, including one small cluster (*n* = 4). Such imbalance is not uncommon in exploratory, data-driven clustering when heterogeneous data contain rare but distinct behavioral patterns. While this small cluster was internally coherent and substantively interpretable, its limited size constrains the stability and generalizability of inferences. Accordingly, the typology should be viewed as exploratory, and future research with larger samples is needed to validate the cluster structure and assess the robustness of rare subgroups*. Finally*, despite high interrater reliability, transforming judicial narratives into categorical variables involves interpretive judgment. Although codebooks and consensus procedures reduced subjectivity, automated text-analysis methods may further enhance transparency and consistency in future research.

## Implications

The differentiated offender patterns identified in this study offer preliminary implications for research, prevention, investigation, and platform governance. Given the exploratory nature of the clustering analysis and dataset limitations, these implications should be viewed as directions for future inquiry rather than definitive guidance for policy, risk assessment, or offender management.

The findings emphasize the need for more refined theoretical models of sextortion and image-based sexual abuse, particularly to account for small but severe subgroups such as 764 extremist network whose behavior cannot be explained by financial motives or image accumulation alone. This group, characterized by sustained harassment, technological intrusion, and profound psychological harm, aligns with growing federal attention to organized online abuse networks (U.S Attorney Office, 2025). Their conduct suggests that existing frameworks emphasizing opportunistic or instrumental offending may be insufficient and that greater attention should be given to domination, control, and thrill-seeking facilitated by online anonymity and asynchronous communication. At the same time, the findings highlight the importance of offender–victim relationship context in interpreting behavioral clusters. Although the analysis captures observable actions, relational dynamics likely shape how coercion is exercised and how harm escalates. Whether victims were strangers, online acquaintances, or former intimate partners may influence the persistence of threats and the trajectory of offending, with limited relational contact lending itself to content-based leverage and repeated interaction enabling more sustained psychological control. While such relational details are inconsistently recorded in judicial documents, recognizing their potential role provides valuable context for understanding variation across clusters and suggests a meaningful direction for future research.

For law enforcement, the observed distinctions across offender subtypes suggest areas where investigative approaches could eventually be differentiated, though further validation is required. Financial sextortion may benefit from future work on detecting scripted monetary demands or monitoring transaction patterns, while CSAM-focused offending emphasizes the importance of cross-jurisdictional coordination and persistent image-based identification. Cases involving deception, hacking, and sustained coercion point to the potential value of rapid-response models linking cybercrime units with victim support services, and hybrid offenders illustrate how integrated cyber–physical investigative approaches may be promising but remain preliminary.

The findings also offer tentative insights for prevention and platform governance. Youth-focused prevention efforts may benefit from emphasizing early recognition of grooming, impersonation, and coercive escalation, particularly given increasing reports of financial sextortion targeting minors. For technology companies, behavioral differences across subtypes suggest possible avenues for strengthening safeguards, such as disrupting repeated monetary solicitations, maintaining robust hashing and identity-matching systems, and exploring detection of behavioral anomalies associated with severe coercion. However, substantial additional research is needed before these approaches can be operationalized.

## Conclusion

This study represents an important step toward empirically mapping the complex and evolving landscape of sextortion offending. Using unsupervised clustering on judicial case data, it reveals the behavioral and motivational diversity of people who offend, and the serious risks posed by certain subtypes, particularly those targeting minors through technological aggression and sustained psychological manipulation. The typology developed here is not merely a statistical classification but an evidence-based framework that captures the varied dynamics of coercion and control underlying these crimes. It demonstrates that sextortion encompasses a continuum of exploitative behaviors, ranging from opportunistic financial manipulation to organized, predatory, and in some cases lethal offenses.

This analysis stresses that sextortion is not a uniform phenomenon but a multifaceted form of digital exploitation that requires tailored responses. By empirically distinguishing subtypes of people who offend through judicial data, the study provides a foundation for developing predictive risk assessment tools, typology-informed sentencing guidelines, and platform-specific intervention protocols. These applications can enhance investigative precision, promote consistency in judicial outcomes, and inform prevention initiatives that account for distinct behaviors of people who offend and victim vulnerabilities.

While the findings acknowledge the significant harm and trauma experienced by victims, particularly young people who often face isolation and silence, they also point to a constructive path forward. Through the systematic identification of patterns among people who offend, this research contributes to a more responsive and accountable system, one capable of transforming empirical insights into practical measures that improve victim protection, guide policy design, and strengthen digital safety in an increasingly interconnected environment.

## Notes

1. Federal judicial documents classify victims only as “minors” without disclosing exact ages for privacy protection. Because specific ages were not available in the source documents, victim age could only be coded as minor versus adult.

2. The diff_value indicates how much a particular feature’s prevalence in a cluster deviate from its overall prevalence in the full sample; a positive value means the feature is more common in that cluster than average, while a negative value means it is less common, helping to identify distinguishing traits of each cluster.
